# Utility of total cell-free DNA levels for surgical damage evaluation in patients with urological surgeries

**DOI:** 10.1038/s41598-021-01430-z

**Published:** 2021-11-11

**Authors:** Sakae Konishi, Takuma Narita, Shingo Hatakeyama, Tohru Yoneyama, Mihoko Sutoh Yoneyama, Yuki Tobisawa, Daisuke Noro, Tendo Sato, Kyo Togashi, Teppei Okamoto, Hayato Yamamoto, Takahiro Yoneyama, Yasuhiro Hashimoto, Chikara Ohyama

**Affiliations:** 1 Department of Urology, Hirosaki University Graduate School of Medicine, Hirosaki, Japan; 2 Department of Advanced Blood Purification Therapy, Hirosaki University Graduate School of Medicine, 5 Zaifu-chou, Hirosaki, 036-8562 Japan; 3 Department of Glycotechnology, Center for Advanced Medical Research, Hirosaki University Graduate School of Medicine, Hirosaki, Japan; 4 Department of Cancer Immunology and Cell Biology, Oyokyo Kidney Research Institute, Hirosaki, Japan; 5 Department of Urology, Mutsu General Hospital, Mutsu, Japan; 6 Department of Urology, Tsugaru General Hospital, Gosyogawara, Japan; 7 Department of Advanced Transplant and Regenerative Medicine, Hirosaki University Graduate School of Medicine, Hirosaki, Japan

**Keywords:** Urogenital diseases, Surgical oncology, Biomarkers

## Abstract

The evaluation of surgical damage is challenging because of the lack of specific biomarkers. Total cell-free DNA (cfDNA) levels have been reported to increase with external trauma and may be a biomarker for tissue damage. To investigate the utility of perioperative total cfDNA levels in evaluating surgical damage in urological surgeries. This multicenter, prospective, observational study included 196 patients scheduled for urological surgeries between September 2020 and July 2021. The primary outcome was the change in total cfDNA levels before and after urological surgery. The secondary outcome was the effect of surgical type on total cfDNA ratio before and after urological surgery. The postoperative median total cfDNA level of the 196 patients was significantly increased 2.5-fold compared to the preoperative level (185.2 ng/mL vs. 406.7 ng/mL, *P* < 0.001). The median total cfDNA before/after ratio was greater than four-fold for kidney transplantation, open cystectomy, and open adrenalectomy. The ratio was less than two-fold for laparoscopic adrenalectomy and robot-assisted radical prostatectomy. Major surgery showed a significant postoperative increase in total cfDNA levels, while minor surgery did not. Total cfDNA levels increased 2.5-fold after urological surgery and it can be used as an acute-phase biomarker for surgical damage.

## Introduction

In recent years, laparoscopic surgery and robot-assisted surgery have become the most common urological procedures^1^. Previous studies have investigated markers for surgical damage, such as operative time, estimated blood loss, length of hospital stay, complications, white blood cell count (WBC), serum C-reactive protein (CRP), cortisol, and cytokines (IL-6, IL-10)^2–4^. However, these are not definitive markers that reflect the hyperacute invasiveness of surgery. The development of sensitive markers is necessary to compare surgical damage.

Plasma cell-free DNA (cfDNA) has recently been of interest as a liquid biopsy for several cancers. Total cfDNA levels are known to increase not only in cancer^5^ but also in pregnancy^6^, sepsis^7^, organ infarction^8^, and trauma^9^. The main cause of cfDNA increase is cell death^10,11^, which has a half-life of approximately 2 h^12^. In the field of trauma therapy, post-injury cfDNA levels and the rate of cfDNA reduction are associated with the severity of trauma and the incidence of complications^9,13–16^. Therefore, the total cfDNA level may be a potential marker of tissue damage in the hyperacute phase. Given that elective surgery is a "planned trauma," total cfDNA level may be a potential biomarker for surgical damage assessment. However, only a few studies have examined the changes in cfDNA levels during the perioperative period of surgical procedures^17,18^, and no studies have evaluated the perioperative total cfDNA level in urological surgeries. The present study aimed to examine the utility of perioperative total cfDNA levels for a surgical damage evaluation in urological surgeries.

## Results

### Stability test of plasma cfDNA at room temperature and 4 °C storage

At room temperature, the total cfDNA decreased two days after blood collection. On the other hand, the total cfDNA was stable for seven days at 4 °C (Fig. S1A). The results of electrophoresis are shown in Fig. S1B, S1C. At room temperature storage, the band intensity around 170 bp becomes weaker after the second day. The background level becomes higher, especially for long fragments. When stored at 4 °C, the band intensity around 170 bp and the background level change was limited.

### Patient demographics and baseline characteristics

**Figure 1:**
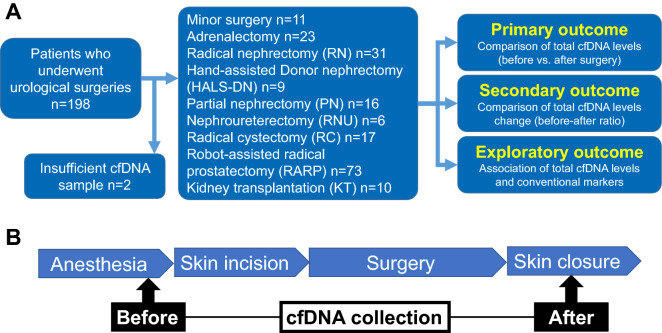
Outline of this study. Outlines of the entire study (**A**) and blood sampling methods (**B**).

Data from 198 Japanese patients who underwent urological surgery at three hospitals were evaluated. Two patients with insufficient cfDNA samples were excluded, and 196 patients were included in the study (Fig. 1).

The median age of patients who underwent urological surgery was 66 years (interquartile range [IQR], 58–72), and 94% of patients were preoperatively assessed as ECOG-PS 0. The most common types of surgery were radical prostatectomy (n = 73), RN (n = 31), adrenalectomy (n = 23), and RC (n = 16) (Table 1). Patient characteristics and surgical outcomes according to procedure are shown in Table 2.

**Table 1:** Background of patients.

n	196
Age, years (IQR)	66 (58–72)
Male, n	153 (78%)
ECOG-PS	
0	183 (94%)
1	11 (5%)
2	2 (1%)
3	0
4	0
Major surgery, n	185 (94%)
Minor surgery, n	11 (6%)
Type of surgery, n	
Radical Prostatectomy	73 (37)
Nephrectomy	
Radical	31 (16)
Partial	16 (8)
Donor	9 (5)
Adrenalectomy	23 (12)
Radical Cystectomy	17 (8)
Kidney transplantation	10 (5)
Radical Nephroureterectomy	6 (3)
TURBT	6 (3)
High orchiectomy	4 (2)
Other	1 (1)

*IQR* interquartile range, *ECOG-PS* Eastern cooperative oncology group performance status, *TURBT* transurethral resection of bladder tumor.

**Table 2:** Patient characteristics and surgical outcomes by type of surgeries.

	n	TNM classification*				Postop. complications				
		T1	T2	T3,4	N1	M1	Surgical time, min (IQR)	Blood loss, g (IQR)	Any grade	Grade ≥ 3
Minor surgery	11	6	2	2	1	0	47 (23–65)	5 (5–5)	0	0
RARP	73	27	12	34	0	4	183 (163–201)	30 (10–50)	11	0
Open radical cystectomy	4	1	1	2	0	0	170	2088	0	0
RARC	13	1	4	8	2	0	357 (316–404)	630 (325–790)	6	1
Open RN	8	3	0	5	0	3	147 (131–170)	145 (28–332)	2	0
Laparoscopic RN	23	18	1	4	0	2	184 (167–203)	20 (5–40)	2	0
HALS-DN	9						217 (23–65)	12 (23–65)	0	0
Open adrenalectomy	4						179 (100–370)	660 (100–1715)	0	0
Laparoscopic adrenalectomy	19						137 (113–154)	5 (5–10)	2	2
RAPN	13	13	0	0	0	0	170 (140–201)	15 (10–50)	0	0
Open partial nephrectomy	3	1	0	0	0	0	155	390		
Kidney transplantation	10						308 (276–370)	190 (125–500)	1	0
Laparoscopic RNU	6	0	2	4	0	0	209 (194–255)	50 (5–73)	1	0

*IQR* interquartile range, *RARP* Robot-assisted radical prostatectomy, *RARC* Robot-assisted radical cystectomy, *RN* Radical nephrectomy, *HALS-DN* Hand-assisted laparoscopic donor nephrectomy, *RAPN* Robot-assisted partial nephrectomy, *RNU* Radical nephroureterectomy.

*TNM classification for patients with non-malignant tumors was not performed.

### Primary outcome: the change in total cfDNA level before and after urological surgery

**Figure 2:**
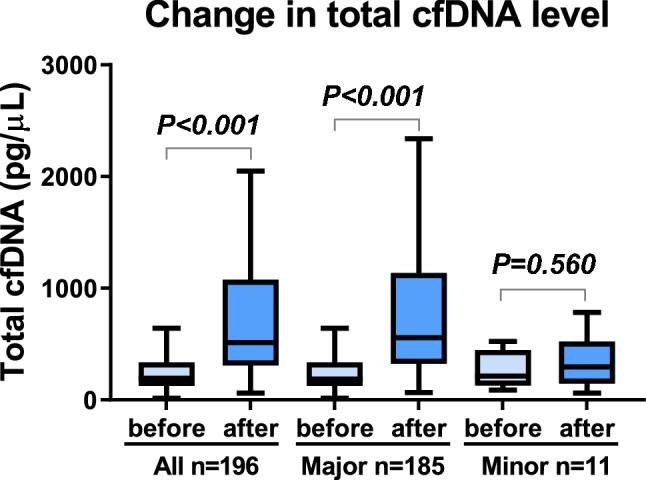
The difference of total cell-free DNA levels before and after surgery. Difference in total cell-free DNA (cfDNA) levels before and after urologic surgery.

As a preliminary study, the trends of total cfDNA levels in major surgeries (robot-assisted radical cystectomy (RARC), RARP, and laparoscopic RN) within four days were evaluated. Results showed that the total cfDNA level increased immediately after surgery and promptly decreased the next day (Fig. S2A). In all eligible patients, the postoperative median total cfDNA levels were significantly increased (Fig. 2, 185.2 ng/mL vs. 406.7 ng/mL, *P* < 0.001), which was 2.5-fold higher than the preoperative level. There was no significant increase in postoperative median total cfDNA levels in patients who underwent minor surgeries (Fig. 2, 214.1 ng/mL vs. 297.2 ng/mL, *P* = 0.560); however, there was a significant increase in total cfDNA levels in most major surgeries. A significant postoperative rise in total cfDNA levels was found in laparoscopic adrenalectomy (Fig. S2B, *P* < 0.001), open RN (Fig. S2C, *P* = 0.008), laparoscopic RN (Fig. S2C, *P* < 0.001), robot-assisted PN (Fig. S2D, *P* < 0.001), robot-assisted RC (Fig. S2E, *P* < 0.001), RARP (Fig. S2F, *P* < 0.001), HALS-DN (Fig. S2G, *P* < 0.001), and KT (Fig. S2H, *P* = 0.004). No significant difference in total cfDNA levels was observed in patients with RNU (Fig. S2I, *P* = 0.18). Statistical difference was not evaluated in open adrenalectomy (Fig. S2B), open PN (Fig. S2D), and open RC (Fig. S2E) because of the small number of patients (n < 5).

### Secondary outcomes: The difference in total cfDNA before/after ratio depending on the type of surgery (open, laparoscopic, or robot-assisted)

**Figure 3:**
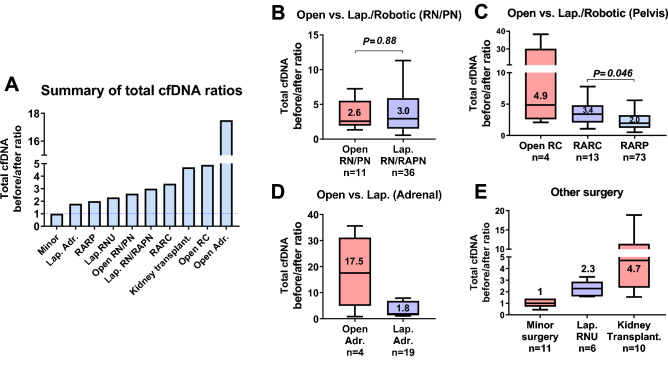
Comparison of total cfDNA ratios before and after surgery for each surgery types. Summary of total cell-free DNA (cfDNA) ratios before and after surgery according to type of surgery (**A**). Comparisons of total cfDNA before/after ratios for open and laparoscopic/robotic kidney surgery (**B**), open and robot-assisted radical cystectomy and robot-assisted radical prostatectomy (**C**), open and laparoscopic adrenalectomy (**D**), and other surgeries (minor surgery, laparoscopic radical nephroureterectomy, kidney transplantation) (**E**).

Figure 3A summarizes the ratios of postoperative total cfDNA levels compared to preoperative levels according to the type of surgery. The median cfDNA before/after ratio was greater than four-fold for KT, open cystectomy, and open adrenalectomy. The ratio was less than two-fold for laparoscopic adrenalectomy and RARP.

The difference in total cfDNA before/after ratio in the same organ according to the type of surgery was then investigated. There was no significant difference in the total cfDNA before/after ratio between open and laparoscopic/robot-assisted surgery of the kidney (Fig. 3B, 2.6-fold vs. 3.0-fold, *P* = 0.88). For pelvic surgery of the prostate and bladder, the total cfDNA before/after ratio of open RC tended to be higher than that of RARP and RARC (Fig. 3C). When RARC and RARP were compared, RARC showed a significantly higher ratio (Fig. 3C, 3.4-fold vs. 2.0-fold, *P* = 0.046). For adrenal surgery, there was a trend towards higher ratios for open surgery (Fig. 3D). The median total cfDNA before and after surgery for other surgeries was 1.0-fold for minor surgery, 2.3-fold for laparoscopic RNU, and 4.7-fold for KT (Fig. 3E).

### Exploratory outcome

**Figure 4:**
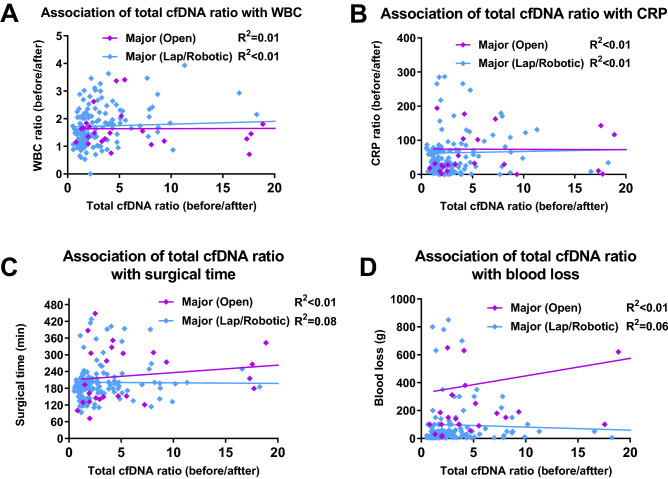
Pearson correlation plots of cell-free DNA ratio before and after surgery with conventional markers. A linear relationship between the cell-free DNA increasing ratio and conventional markers by scatterplot and linear regression analysis with correlation coefficient R. Markers examined were WBC ratio (before and after surgery) (**A**), CRP ratio (before and after surgery) (**B**), surgical time (**C**) and blood loss (**D**).

There was no significant correlation between the postoperative increase ratio of WBC (Fig. 4A), increase ratio of CRP (Fig. 4B), surgical time (Fig. 4C), and estimated blood loss (Fig. 4D) with total cfDNA before/after ratio, regardless of the type of surgery. There was also no correlation between the conventional surgical damage markers and postoperative total cfDNA levels.

Total cfDNA before/after ratios were not significantly associated with or without postoperative complications for RARP (Fig. S3A) and RARC (Fig. S3B).

## Discussion

The current study is the first to investigate the utility of perioperative total cfDNA levels for the evaluation of surgical damage in urological surgeries. Previous study have used to assess the extent of damage in the trauma field^9,13–16^. A review article examining the utility of post-traumatic cfDNA measurement in 904 patients from 14 observational studies found a correlation between total cfDNA level and severity of injury, post-traumatic complications, mortality, and length of stay in the intensive care unit^9^. However, a strong limitation of these trauma studies is the lack of pre-traumatic cfDNA levels. The review article also commented that cfDNA should be examined pre- and post-surgery to assess the extent of damage^9^. Accordingly, the current study evaluated the pre- and postoperative values of total cfDNA for elective surgeries. Results showed that the postoperative total cfDNA level was significantly higher than the preoperative level (Fig. 1). The median total cfDNA before/after ratio tended to be higher in open surgeries and was almost the same value in minor surgeries. As cfDNA is presumed to be released by cell injury, necrosis, or inflammation^10,11^, total cfDNA levels might be helpful in evaluating surgical damage.

The utility of total cfDNA levels for the assessment of surgical damage needs further discussion. Only one previous article on the changes in total cfDNA levels pre- and post-surgery is present. A prospective exploratory study of 10 patients undergoing arthroscopic surgery (orthopedic knee replacement surgery, n = 5; orthopedic hip replacement surgery, n = 5) reported that the median total cfDNA levels increased immediately after surgery and decreased on the first postoperative day^17^. These results were similar to those of the current study, and total cfDNA levels might be useful to assess external damage as an acute-phase biomarker. However, there is the lack of available information regarding the source of cfDNA, which is speculated to be released from damaged cells and immune cells. Therefore, it is difficult to conclude that the total cfDNA level is a useful biomarker for surgical damage. Further studies are necessary to address its role in the tissue damage.

Results showed a difference in total cfDNA levels between open and laparoscopic/robotic surgeries (Fig. 3A). The total cfDNA increase ratio was minimal for minor surgery, moderate for laparoscopic/robot-assisted surgery, and maximal for open surgery. This trend may represent surgical damage, reflecting the number of cell deaths. First, a small skin incision might be a key factor for surgical damage, and open surgery may have a higher total cfDNA level. However, nephrectomy showed no significant difference in the total cfDNA before/after ratio between the open and laparoscopic/robotic surgeries (Fig. 3B). This result may suggest similar tissue damage in kidney surgery regardless of the procedure. The similar perirenal peeling area might be the reason, but little is known about the effect of skin incision or peeling on total cfDNA levels. Second, small cases of open RN/PN under different incisions (abdominal midline incision or oblique lumbar incision) are limitations of this study. Further studies are required to answer these questions.

The researchers speculated that surgical damage can be associated with conventional parameters such as postoperative WBC count, CRP level, surgical time, and blood loss. However, no relationship was found between total cfDNA before/after ratio in major surgery and these parameters (Fig. 4). These findings suggest that total cfDNA levels may have different properties than inflammatory markers, such as WBC and CRP. As the total cfDNA rises and falls rapidly, it might be associated with cortisol and cytokines (IL-6 and IL-10). Several studies have suggested a potential relationship between surgical stress and cortisol/cytokines^2–4^. Future investigations should address the potential role of cortisol, IL-6, and total cfDNA levels in surgical damage.

The association between total cfDNA level and postoperative complications is the object of interest. As a previous study showed a positive association between the total cfDNA level and post-traumatic complications^13,19^, elevated postoperative total cfDNA levels may be related to a higher rate of postoperative complications. However, results showed that the total cfDNA before/after ratio was not significantly different between patients with and without postoperative complications (Fig. S3). As the total cfDNA level falls rapidly after surgery, continuous measurements might be more useful than single point analysis to predict postoperative events. The small number of events (n = 3 in grade ≥ 3) was the major limitation of this study, and further study is needed to investigate the utility of total cfDNA level on predicting postoperative complications.

This study has several limitations. First, a small number of cases could be a source of bias. In particular, the number of patients undergoing open surgery was significantly lower owing to the recent trend of minimally invasive surgery. However, this is the first study to investigate perioperative total cfDNA levels and its usefulness in urological surgeries. Further large-scale validation studies and investigation of the relationship between cfDNA levels and surgical outcomes are needed.

In conclusion, total cfDNA levels increased 2.5-fold after urological surgery. The cfDNA increase ratio was minimal for minor surgery, moderate for laparoscopic/robot-assisted surgery, and maximal for open surgery. Total cfDNA levels may be a new acute-phase biomarker for surgical damage. Further studies are required.

## Methods

We performed a multicenter, prospective, observational study in accordance with the ethical standards outlined in the Declaration of Helsinki. The ethics committee of the Hirosaki University School of Medicine approved this study at Hirosaki University School of Medicine, Mutsu general hospital and Tsugaru general hospital (authorization number: 2020–258). Written consents were obtained from all participants.

### Study population and patient selection

An outline of this study is shown in Fig. 1A. Patients scheduled for urological surgeries at one academic and two satellite centers between September 2020 and July 2021 were evaluated. The urological surgeries included in this study were transurethral resection of the bladder tumor (TURBT), high orchiectomy, adrenalectomy, radical nephrectomy (RN), hand-assisted laparoscopic donor nephrectomy (HALS-DN), partial nephrectomy (PN), radical nephroureterectomy (RNU), radical cystectomy (RC), robot-assisted radical prostatectomy (RARP), and kidney transplantation (KT). TURBT and high orchiectomy were defined as minor surgeries, while the rest were defined as major surgeries. Patients with insufficient cfDNA were excluded. The selection of open, laparoscopic, or robot-assisted surgery was discussed at the meeting in the researchers’ department prior to surgery.

### Variable evaluations

The following parameters of patients were analyzed: age, sex, Eastern Cooperative Oncology Group performance status (ECOG-PS), history of neoadjuvant therapy, operation time, intraoperative estimated blood loss, postoperative peripheral blood data, serum albumin, and CRP. Tumor stage was assigned based on the 2009 TNM classification of the Union for International Cancer Control. Postoperative complications were evaluated using the Clavien-Dindo classification.

### Blood Sampling and Plasma preparation

Blood (7 cc) was collected from the radial arterial line using a BD Vacutainer® Barricor™ plasma blood collection tube with heparin lithium prior to surgery (before skin incision) and at the end of surgery (at skin closure) (Fig. 1B). In some patients (n = 10), peripheral venous blood was collected on postoperative days one and four to confirm the trend of total cfDNA levels. The blood was centrifuged at 3000 g for 10 min immediately after blood collection, and total cfDNA levels were either measured immediately or the centrifuged blood was stored at 4 °C.

### Stability test of plasma cfDNA at room temperature and 4 °C storage

We investigated the stability of the total cfDNA. Each of the three plasma samples was divided into six aliquots and stored at room temperature and 4 °C. We performed electrophoresis of each blood sample on day 0 (immediately), 1, 2, 3, 6 and, 7 to examine the changes in cfDNA. Also, the electropherogram at day 0, 3, and 7 were evaluated.

### Plasma cfDNA extraction and cfDNA characteristics

The AB MagMAX Cell-Free DNA Isolation Kit (Applied Biosystems, Foster City, CA, USA) was used to extract cfDNA from the plasma sample (1 mL). The total cfDNA levels were analyzed using an Agilent High Sensitivity DNA Kit and Agilent Bioanalyzer 2100 (Agilent Technologies Japan, Ltd., Tokyo, Japan). The detection limit of cfDNA in this experimental system is 5 pg/μL, and the fragment length can be measured from 50 to 7000 bp. We measured a fragment of the mono-nucleosomes around 170 bp. The range of fragment length and the calculation of cfDNA concentrations were determined automatically by 2100 Expert Software ver. B.02.08.SI648(SR2) (Aglient Technologies, Santa Clara, United States). Two experimenters in a single institution (Hirosaki University) analyzed the total cfDNA levels based on the same protocol.

### Outcome measurements

The primary outcome was the change in total cfDNA levels before and after urological surgery, and the secondary outcome was the difference in total cfDNA before/after ratio, depending on the type of surgery. The exploratory outcomes were the association of total cfDNA before/after ratio and conventional markers before operation/after operation ratio (white blood cell counts, CRP, operation time, and estimated blood loss) and the association of total cfDNA before/after ratio and postoperative complications. White blood cell counts and CRP data were taken 1 h and 12–18 h after the surgery, respectively.

### Statistical analysis

Statistical analyses of clinical data were performed using BellCurve for Excel (Social Survey Research Information Co., Ltd., Tokyo, Japan) and GraphPad Prism v. 9.12 (GraphPad Software, San Diego, CA, USA). Categorical variables were reported as percentages and compared using Fisher’s exact test. Quantitative data were expressed as medians with quartiles. Differences between the groups were statistically compared using the Mann–Whitney U test. Statistical significance was set at *P* < 0.05. A linear relationship between the two variables was evaluated by scatterplot and linear regression analysis with correlation coefficient R. Absolute R values of 0.00–0.24, 0.25–0.49, 0.50–0.74, and 0.75–1.00 were defined as none to very weak, weak, moderate, and strong linear relationships, respectively.

### Consent for Publication

All authors approved for the publication.

## Supplementary Information

Supplementary Information 1.

Supplementary Information 2.

## Data Availability

The minimal data set of the present study is available on request.

## References

[1] Rassweiler JJ, Teber D (2016). Advances in laparoscopic surgery in urology. Nat. Rev. Urol..

[2] Miyake H (2002). Comparison of surgical stress between laparoscopy and open surgery in the field of urology by measurement of humoral mediators. Int. J. Urol..

[3] Porcaro AB (2015). Robotic-assisted radical prostatectomy is less stressful than the open approach: results of a contemporary prospective study evaluating pathophysiology of cortisol stress-related kinetics in prostate cancer surgery. J. Robot. Surg..

[4] Rubinstein, M. *et al.* Prospective, randomized comparison of transperitoneal versus retroperitoneal laparoscopic adrenalectomy. *J. Urol.* **174**, 442–5; discussion 445 (2005). 10.1097/01.ju.0000165336.44836.2d 16006861

[5] Corcoran RB, Chabner BA (2018). Application of cell-free DNA analysis to cancer treatment. N. Engl. J. Med..

[6] Bianchi DW, Chiu RWK (2018). Sequencing of circulating cell-free DNA during pregnancy. N. Engl. J. Med..

[7] Dwivedi DJ (2012). Prognostic utility and characterization of cell-free DNA in patients with severe sepsis. Crit. Care.

[8] Chang CP-Y (2003). Elevated cell-free serum DNA detected in patients with myocardial infarction. Clin. Chim. Acta.

[9] Gögenur M, Burcharth J, Gögenur I (2017). The role of total cell-free DNA in predicting outcomes among trauma patients in the intensive care unit: a systematic review. Crit. Care.

[10] Fournié GJ (1995). Plasma DNA as a marker of cancerous cell death. Investigations in patients suffering from lung cancer and in nude mice bearing human tumours. Cancer Lett..

[11] Fournié GJ, Martres F, Pourrat JP, Alary C, Rumeau M (1993). Plasma DNA as cell death marker in elderly patients. Gerontology.

[12] Diehl F (2008). Circulating mutant DNA to assess tumor dynamics. Nat. Med..

[13] Lo YM, Rainer TH, Chan LY, Hjelm NM, Cocks RA (2000). Plasma DNA as a prognostic marker in trauma patients. Clin. Chem..

[14] Lam NYL, Rainer TH, Chan LYS, Joynt GM, Lo YMD (2003). Time course of early and late changes in plasma DNA in trauma patients. Clin. Chem..

[15] Macher H (2012). Role of early cell-free DNA levels decrease as a predictive marker of fatal outcome after severe traumatic brain injury. Clin. Chim. Acta.

[16] Ren B (2013). Is plasma cell-free DNA really a useful marker for diagnosis and treatment of trauma patients?. Clin. Chim. Acta.

[17] Brodbeck K (2019). Quantitative analysis of individual cell-free DNA concentration before and after penetrating trauma. Int. J. Legal Med..

[18] Henriksen TV (2020). The effect of surgical trauma on circulating free DNA levels in cancer patients-implications for studies of circulating tumor DNA. Mol. Oncol..

[19] Rainer TH (2001). Plasma DNA, prediction and post-traumatic complications. Clin. Chim. Acta.

