# Supplementary material for: Utility of total cell-free DNA levels for surgical damage evaluation in patients with urological surgeries
Source: Sci Rep. 2021 Nov 11;11:22103. doi: 10.1038/s41598-021-01430-z (PMC8585863; doi:10.1038/s41598-021-01430-z)
Supplement: Supplementary file 1 — Supplementary Information 1. [file 41598_2021_1430_MOESM1_ESM.docx]

**Supplementary Fig.1 Stability of total cfDNA in vitro at room temperature and at 4°C storage.**

Comparison of the stability of total cfDNA concentration up to day 7 at room temperature and 4°C storage (**A**). Time course of cfDNA gel image of electrophoresis on day 0 (immediately), 1, 2, 3, 6 and, 7 stored at room temperature and at 4°C (**B**). The electropherogram at day 0, 3, and 7 stored at room temperature and at 4°C (**C**).

**Supplementary Fig.2 The difference of total cell-free DNA levels before and after surgery by type of surgery.**

Trend of total cfDNA levels in ten patients up to postoperative day four (**A**). Comparisons of total cell-free DNA levels before and after surgery for open and laparoscopic adrenalectomy (**B**), open and laparoscopic radical nephrectomy (**C**), open and robot-assisted partial nephrectomy (**D**), open and robot-assisted radical cystectomy (**E**), robot-assisted partial prostatectomy (**F**), hand-asisted laparoscopic donor nephrectomy (**G**), kidney transplantation (**H**), and laparoscopic nephroureterectomy (**I**) are shown.

**Supplementary Fig.3 Comparison of total cfDNA before/after ratios with and without complications for each surgery types.**

Comparisons of total cfDNA before/after ratios with and without postoperative complications for robot-assisted radical prostatectomy (**A**) and robot-assisted radical cystectomy (**B**).
