# Supplementary material for: Utility of total cell-free DNA levels for surgical damage evaluation in patients with urological surgeries
Source: Sci Rep. 2021 Nov 11;11:22103. doi: 10.1038/s41598-021-01430-z (PMC8585863; doi:10.1038/s41598-021-01430-z)
Supplement: Supplementary file 2 — Supplementary Information 2. [file 41598_2021_1430_MOESM2_ESM.pdf]

**Fig.S1**

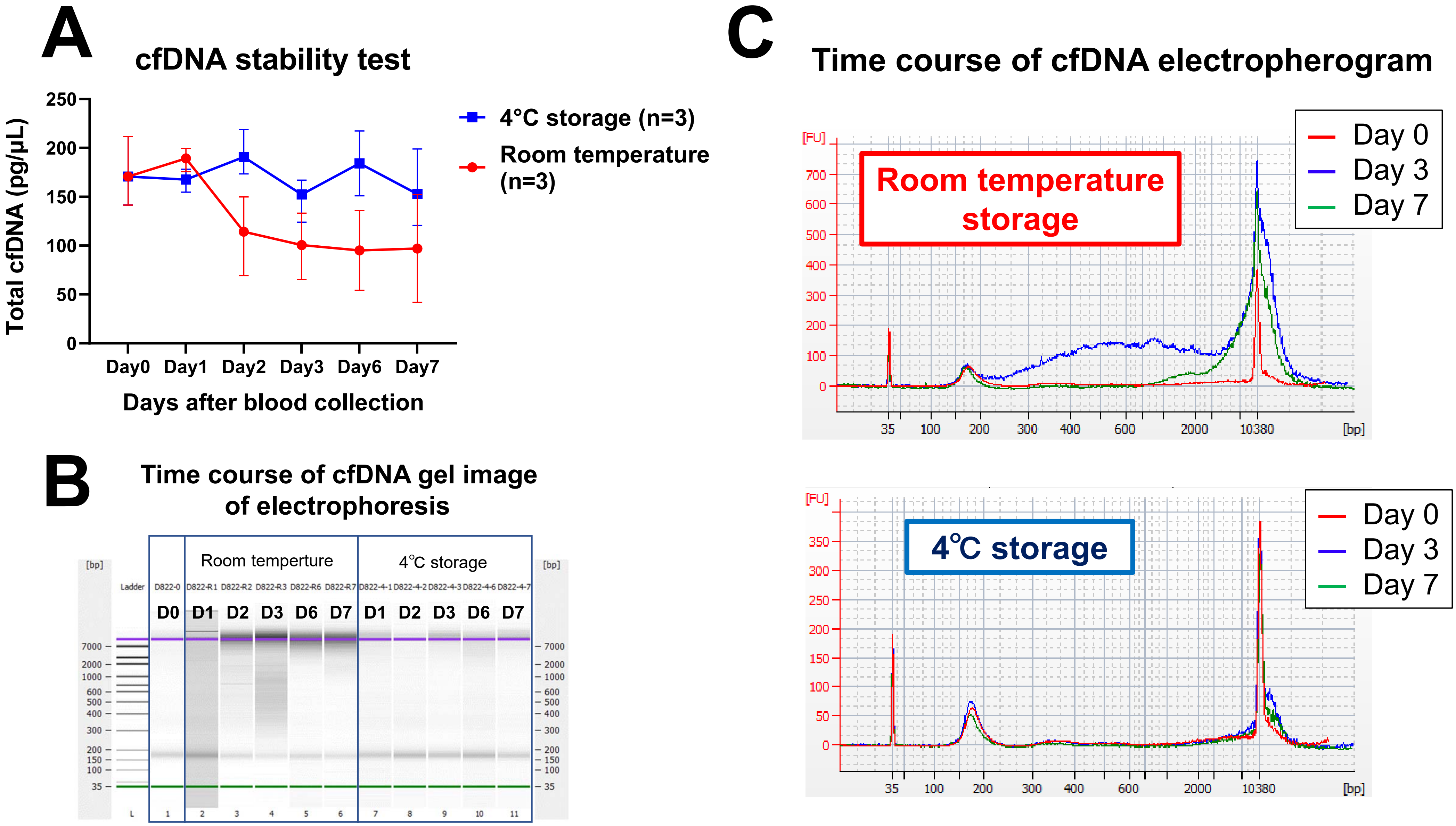
A
cfDNA stability test
Total cfDNA (pg/μL)
250
200
150
100
50
0
Day0
Day1
Day2
Day3
Day6
Day7
Days after blood collection
4°C storage (n=3)
Room temperature (n=3)
B
Time course of cfDNA gel image of electrophoresis
[bp]
Ladder
D822-0
Room temperture
D822-R1 D822-R2 D822-R3 D822-R6 D822-R7
4℃ storage
D822-4-1 D822-4-2 D822-4-3 D822-4-6 D822-4-7
D0 D1 D2 D3 D6 D7 D1 D2 D3 D6 D7
7000
2000
1000
600
500
400
300
200
150
100
35
L 1 2 3 4 5 6 7 8 9 10 11
C
Time course of cfDNA electropherogram
Room temperature storage
[FU]
700
600
500
400
300
200
100
0
35 100 200 300 400 600 2000 10380 [bp]
Day 0
Day 3
Day 7
4℃ storage
[FU]
350
300
250
200
150
100
50
0
35 100 200 300 400 600 2000 10380 [bp]
Day 0
Day 3
Day 7

Fig.S2

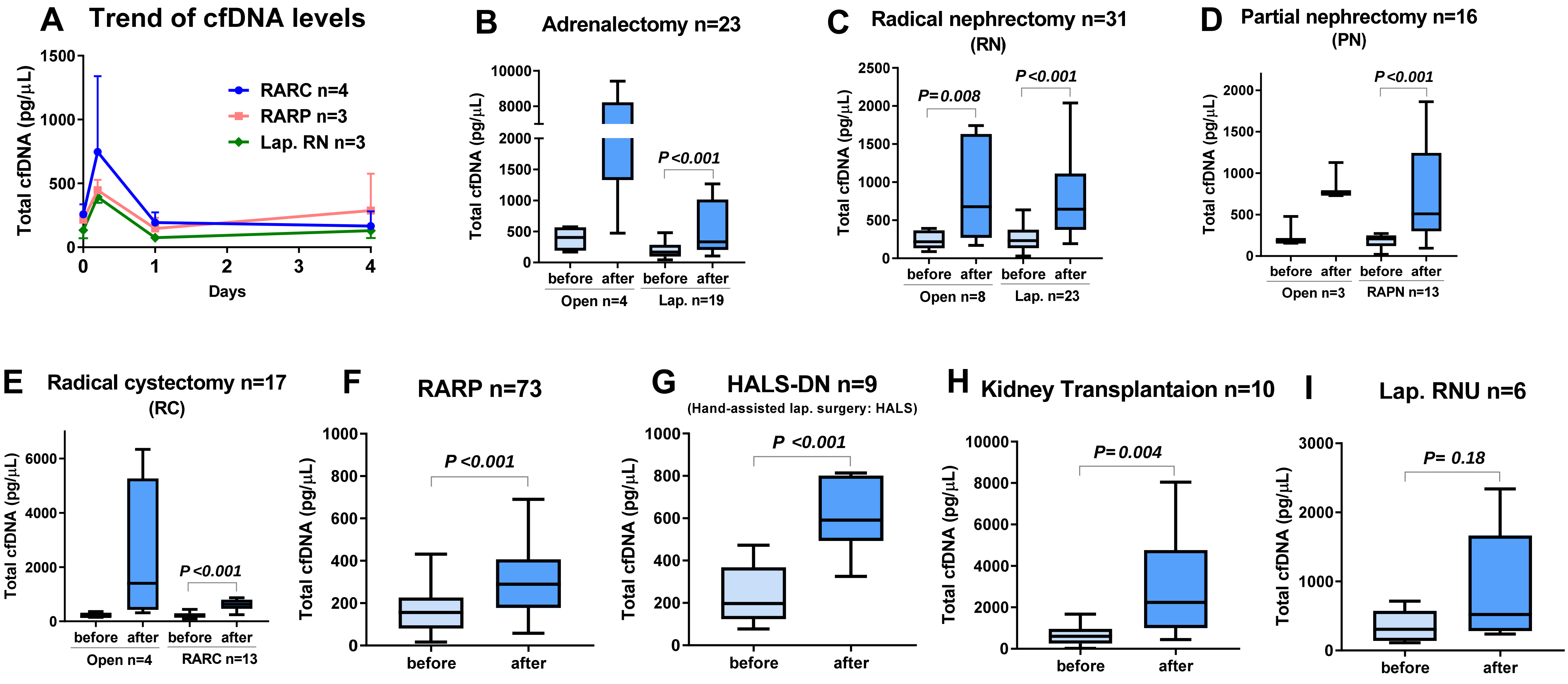
A
Trend of cfDNA levels
Total cfDNA (pg/μL)
Days
RARC n=4
RARP n=3
Lap. RN n=3
B
Adrenalectomy n=23
before
after
Open n=4
Lap. n=19
P<0.001
C
Radical nephrectomy n=31
(RN)
P=0.008
P<0.001
Open n=8
Lap. n=23
D
Partial nephrectomy n=16
(PN)
P<0.001
Open n=3
RAPN n=13
E
Radical cystectomy n=17
(RC)
P<0.001
Open n=4
RARC n=13
F
RARP n=73
P<0.001
G
HALS-DN n=9
(Hand-assisted lap. surgery: HALS)
P <0.001
H
Kidney Transplantaion n=10
P= 0.004
I
Lap. RNU n=6
P= 0.18

Fig.S3

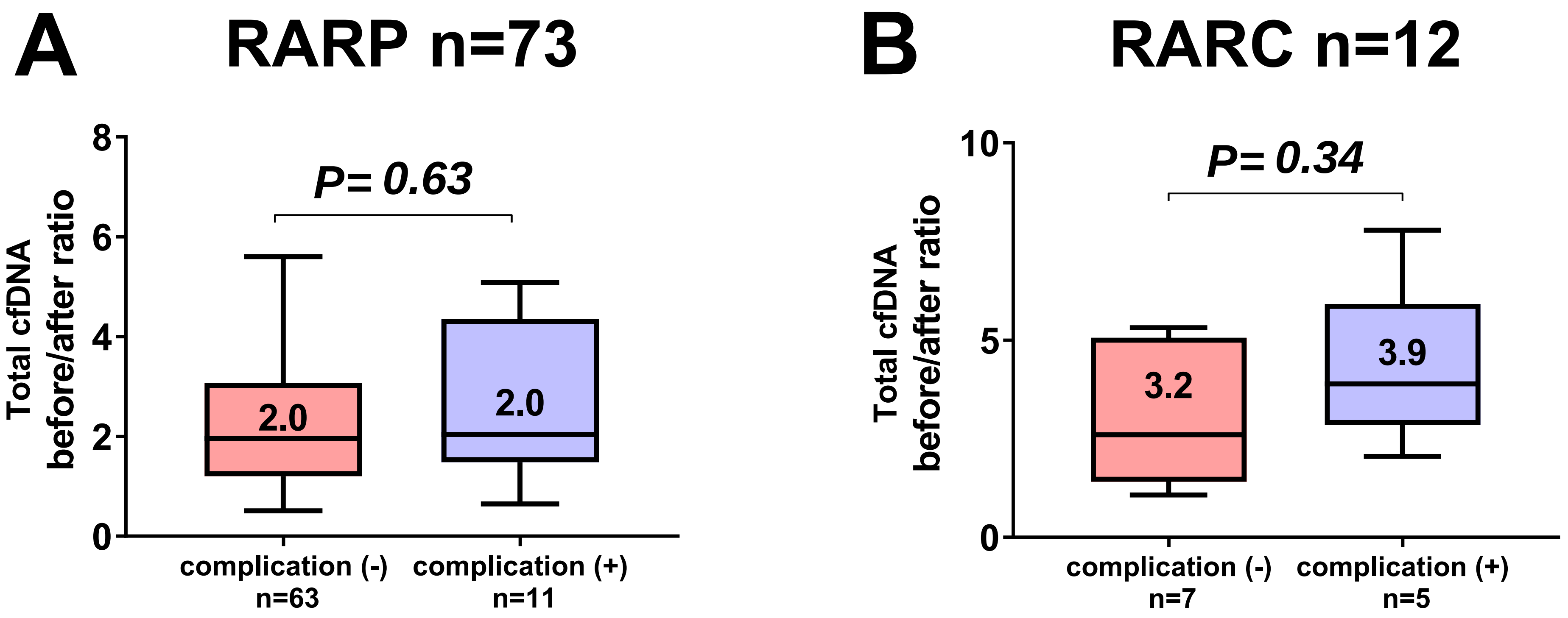
A
RARP n=73
Total cfDNA before/after ratio
8
6
4
2
0
P= 0.63
2.0
2.0
complication (-) n=63
complication (+) n=11
B
RARC n=12
Total cfDNA before/after ratio
10
5
0
P= 0.34
3.2
3.9
complication (-) n=7
complication (+) n=5
